# A Revision of the Genus *Argolis* (Hemiptera: Reduviidae: Stenopodainae) from Asia †

**DOI:** 10.3390/insects14080680

**Published:** 2023-07-31

**Authors:** Zhuo Chen, Michael D. Webb, Wanzhi Cai

**Affiliations:** 1 Department of Entomology and MOA Key Lab of Pest Monitoring and Green Management, College of Plant Protection, China Agricultural University, Yuanmingyuan West Road, Beijing 100193, China; 2 Department of Collections (Insects), The Natural History Museum, Cromwell Road, London SW7 5BD, UK

**Keywords:** Heteroptera, lectotype, new combination, new synonymy, sexual dimorphism, Oriental Realm, Palaearctic Realm

## Abstract

**Simple Summary:**

The assassin bug subfamily Stenopodainae is the fifth largest group of Reduviidae, with about 770 valid species known worldwide. Many taxa of Stenopodainae have been poorly studied and are therefore in need of revision. *Argolis* Stål, 1861 is a medium-sized genus of Stenopodainae with native species occurring in Sub-Saharan Africa and Asia. The Asian fauna of *Argolis* is revised in this study, resulting in the recognition of two species, and the Oriental genera *Bardesanes* Distant, 1909 and *Neoklugia* Distant, 1919 are here considered junior synonyms of *Argolis*. Taxonomic changes are proposed accordingly in the present study, and the significant sexual dimorphism, systematic relationships, and distribution of *Argolis* are also discussed.

**Abstract:**

The assassin bug genus *Argolis* Stål, 1861 (Hemiptera: Reduviidae: Stenopodainae) has a disjunct distribution in Sub-Saharan Africa and Asia. In the present study, the Asian species of *Argolis* are revised. Two species are recognized, redescribed, and illustrated, with the following new subjective synonyms and new combination proposed: *Argolis* Stål, 1861 = *Bardesanes* Distant, 1909, **syn. nov.** = *Neoklugia* Distant, 1919, **syn. nov.**; *A. farinator* (Reuter, 1882) = *N. typica* Distant, 1919, **syn. nov.** = *B. sericenotatus* Livingstone & Ravichandran, 1989, **syn. nov.**; *A. signata* (Distant, 1909), **comb. nov.** (transferred from *Bardesanes*) = *Caunus noctulus* Hsiao, 1977, **syn. nov.** Lectotypes for *C. farinator*, *B. signatus*, and *N. typica* are designated. A key to separate the two Asian species of *Argolis* is provided. The sexual dimorphism, systematic relationships, and distribution of *Argolis* are discussed. *Argolis* is newly recorded from Laos, Pakistan, and Vietnam.

## 1. Introduction

The assassin bug subfamily Stenopodainae is a large but relatively less researched group of Reduviidae, comprising 112 described genera and approximately 770 described species [1,2,3]. Some genera are speciose and widely distributed, such as the cosmopolitan *Oncocephalus* Klug, 1830 (>200 spp.) and *Pygolampis* Germar, 1817 (91 spp.) and the Old World *Sastrapada* Amyot & Serville, 1843 (71 spp.). However, 52 genera of Stenopodainae are monotypic, and most of them lack sufficient descriptions and/or illustrations, which makes their identity questionable and, therefore, largely impedes our understanding of relationships within the subfamily. Our previous studies have reviewed some genera of Stenopodainae from Asia [4,5], aiming to increase our knowledge of the diverse Asian stenopodaine fauna. The present study is another part of this topic, revising the Asian fauna of the genus *Argolis* Stål, 1861.

*Argolis*, more commonly known as *Caunus* Stål, 1865 in the past few decades, currently contains 16 described species, with its highest diversity occurring in the Afrotropical Realm (14 species), while two species, *A. farinator* (Reuter, 1882) and *A. noctula* (Hsiao, 1977), are distributed in South and East Asia [1,6]. Members of *Argolis* are known to display significant sexual dimorphism [6,7,8], resulting in the male and female of this genus being originally described as separate genera [9,10,11]. The species-level classification of this genus also needs to be revised, as many species were originally described from a single sex [12,13,14,15], but little new information has since been published.

In the present study, we review the Asian species of *Argolis* as well as those of two similar genera, *Bardesanes* Distant, 1909 and *Neoklugia* Distant, 1919, and consider them all congeneric, with *Argolis* as the senior name. These and other nomenclatorial changes are made, and the sexual dimorphism, systematic relationships, and distribution of *Argolis* are also discussed.

## 2. Materials and Methods

Specimens examined or cited in the present study are deposited in the following institutions:
BMNHThe Natural History Museum, London, UKBUCIBharathiar University, Coimbatore, IndiaCAUEntomological Museum, China Agricultural University, Beijing, ChinaIZASInstitute of Zoology, Chinese Academy of Sciences, Beijing, ChinaMNHNMuséum National d’Histoire Naturelle, Paris, FranceNIAESNational Institute for Agro-Environmental Sciences, Tsukuba, JapanZMUCNatural History Museum of Denmark, University of Copenhagen, Copenhagen, Denmark

Label data of type specimens are copied verbatim in quotation marks (“ ”); lines on the same label are separated by a backslash (\); different labels are separated by a semicolon (;); and comments on label data are provided in square brackets ([ ]); printed (pr.) and handwritten (hw.) texts are indicated.

Male and female genitalia were soaked in a heated 10% KOH solution for approximately ten minutes to remove soft tissue, rinsed in distilled water, and dissected under a Nikon SMZ745 stereoscopic microscope. Dissected genitalia were placed in a vial containing glycerin and, after examination, pinned under the corresponding specimen. For the construction of the redescription of *Argolis*, specimens of some Afrotropical species of the genus were examined.

Photographs were taken using a Canon 7D Mark II digital camera with a Canon micro lens EF 100 mm and MP-E 65 mm for habitus, and a Zeiss Axio Zoom. V16 microscope for dissected body parts. Helicon Focus version 5.3 was used for image stacking. Figures were assembled using Adobe Photoshop 2020. The distribution map was prepared using the online version of SimpleMappr [16].

Morphological terminology mainly follows Weirauch [17]. Measurements were obtained using a calibrated micrometer. Distributional data are given from specimens examined in this study and literature records; new distributional records are marked by an asterisk (*); sources of data from literature records (without specimen examination) are given.

## 3. Results

### 3.1. Argolis Stål, 1861

(Figure 1, Figure 2, Figure 3, Figure 4, Figure 5, Figure 6, Figure 7, Figure 8 and Figure 9)

*Argolis* Stål, 1861: 146 [9] (protologue); Stål (1865: 150, 153) [10] (in key, redescription, Afrotropical); Walker (1873a: 79) [18] (in key); Walker (1873b: 26) [19] (as subgenus of *Oncocephalus* Klug, 1830); Stål (1874: 89) [20] (catalogue); Lethierry and Severin (1896: 89) [21] (catalogue); Jeannel (1919: 167, 173) [22] (in key, distribution, fauna of East Africa); Schouteden (1931: 112) [11] (listed, fauna of the Democratic Republic of the Congo); Villiers (1948: 365, 405) [7] (in key, redescription, distribution, Afrotropical); Villiers (1968: 106, 173) [8] (in key, redescription, distribution, fauna of Madagascar); Putshkov (1985: 14) [23] (nomenclature); Swanson (2018: 179) [24] (nomenclature). **Type species** by original designation: *Oncocephalus calabarensis* Stål, 1858.

*Caunus* Stål, 1865: 150, 153 [10] (protologue); Walker (1873a: 78) [18] (in key); Walker (1873b: 31) [19] (as subgenus of *Stenopoda* Laporte, 1833); Stål (1874: 84, 87) [20] (in key, catalogue); Reuter (1882: 750) [12] (redescription); Lethierry and Severin (1896: 89) [21] (catalogue); Schouteden (1902: 242) [25] (as subgenus of *Argolis*); Distant (1903: 222, 233) [26] (in key, redescription, distribution, fauna of India, Sri Lanka and Myanmar); Jeannel (1919: 167, 174) [22] (in key, distribution, fauna of East Africa); Hsiao (1977: 68, 74) [27] (in key, listed, fauna of China); Hsiao and Ren (1981: 465, 477) [28] (in key, listed, fauna of China); Putshkov (1985: 14) [23] (nomenclature); Maldonado-Capriles (1990: 497) [1] (catalogue); Livingstone and Ravichandran (1991: 28) [29] (in key, fauna of southern India); Putshkov and Putshkov (1996: 209) [30] (catalogue, Palaearctic); Gupta et al. (2005: 130) [31] (catalogue, distribution, fauna of India); Ambrose (2006: 2405) [32] (listed, fauna of India); Cao et al. (2011: 50) [6] (diagnosis, distribution); Ishikawa and Miyamoto (2012: 276, 277) [33] (in key, diagnosis, distribution, fauna of Japan); Aukema et al. (2013: 127) [34] (listed, Palaearctic); Ishikawa (2016: 449) [35] (listed, fauna of Japan); Swanson (2018: 179) [24] (nomenclature). Type species by monotypy: *Stenopoda capensis* Stål, 1855. Synonymized by Schouteden (1931: 112) [11].

*Bardesanes* Distant, 1909: 363 [36] (protologue); Distant (1910: 187) [37] (redescription, distribution, fauna of India, Sri Lanka and Myanmar); Maldonado-Capriles (1990: 495) [1] (catalogue); Livingstone and Ravichandran (1991: 27) [29] (in key, fauna of southern India); Gupta et al. (2005: 132) [31] (catalogue, distribution, fauna of India); Ambrose (2006: 2405) [32] (listed, fauna of India). Type species by monotypy: *Bardesanes signatus* Distant, 1909. **New subjective synonym.**

*Neoklugia* Distant, 1919: 71 [38] (protologue); Maldonado-Capriles (1990: 508) [1] (catalogue); Ambrose (2006: 2405) [32] (listed, fauna of India). Type species by monotypy: *Neoklugia typica* Distant, 1919. **New subjective synonym.**

**Diagnosis.** Recognized within Stenopodainae by the following combination of characters: head subcylindrical with anteocular region more than two times as long as postocular; mandibular plates short but sharply produced anteriorly; eye reniform in lateral view; labium nearly straight, with visible segments I and II subequal in length; anterior lobe of pronotum with four small granulations arranged squarely on disc (sometimes indistinct); fore femur slender, lacking ventral spines or denticles; tarsal formula 3-3-3; hemelytron with hexagonal cubital cell. Only the macropterous form is known.

**Redescription.** Macropterous male (Figure 2A–C and Figure 4A–C). **Vestiture.** Body surface dull, finely rugose, densely covered with tiny, decumbent, scale-like setae arising from small wart-like tubercles on head, thorax, legs, veins of coriaceous portion of hemelytron, and abdomen. Head with short, decumbent, whitish pubescence; postocular region and lateroventral margin of antocular region with several distinct setigerous tubercles (Figure 5A,B,E,F); antennal scape and pedicel with long, erect, thick setae on dorsal and lateral surfaces and long, erect, slender setae on ventral surface; flagellomeres and apex of pedicel with shorter suberect setae intermixed with short, decumbent, whitish pubescence; labium with sparse tiny setae. Pronotum, lateral sides of prosternal grove, anterodorsal margin of mesopleuron, mesosternum, dorsal margin of metapleuron, and anterior margin of metasternum with very short, decumbent, whitish pubescence; pronotum with several narrow, longitudinal, glabrous areas on disc (Figure 5A,E). Legs with short to long, decumbent to suberect, thick setae arising from small tubercles. Abdominal sternite II with dense, short, decumbent, whitish pubescence; lateral and posterior margins of segment VII with short scale-like setae.

**Structure.** Body elongate oval (Figure 2A–C and Figure 4A–C). Head (Figure 5A,B,E,F) elongate, subcylindrical, shorter than pronotum; anteocular region more than two times as long as postocular, nearly parallel-sided in dorsal view; mandibular plates short, slightly to distinctly divergent anteriorly, with sharp apices; antennifer with small setigerous lateral process; postocular region gradually convergent posteriorly, with deep longitudinal sulcus between ocelli and one pair of tubercles on posterior head margin. Eye (Figure 5A,B,E,F) large, strongly protruding laterally, reniform in lateral view, dorsal margin remote from dorsal head margin and ventral margin distinctly surpassing ventral head margin, ventromedial margin nearly touching each other. Ocellus (Figure 5A,B,E,F) large, elevated, protruding laterodorsally. Antennal scape thickest, distinctly longer than head, weakly curved near apex; pedicel longest, slightly curved; flagellum short, gracile, distiflagellomere about two times as long as basiflagellomere. Labium (Figure 5B,F) nearly straight, tapering; visible segments I and II subequal in length; visible segment III shortest. Collum distinctly distinguished from postocular region of head.

Pronotum (Figure 5A,B,E,F) subtrapezoidal, length along midline slightly shorter than width across humeral angles; anterior margin slightly concave; anterolateral angles blunt, angulated or acute, usually projecting anterolaterally; anterior pronotal lobe distinctly shorter than posterior lobe, with median depression on posterior half and four small granulations arranged squarely on disc (sometimes indistinct); posterior pronotal lobe separated from anterior lobe by faint transverse sulcus, with shallow medial longitudinal furrow; lateral margin finely concave; humeral angles acute, slightly or distinctly protruding laterally; posterior margin oblique on lateral third, nearly straight or finely concave on middle third. Anterolateral angles of prosternum forming small acute processes (Figure 5B,F); fore acetabulum open. Scutellum (Figure 5A,B) elongate triangular, with one pair of small basolateral denticles; apical process narrow and acute, curved upwards.

Legs slender. Fore femur simple, lacking ventral spines or denticles; fore tibia straight, with tibial comb at apex of inner surface. Mid tibia slightly longer than mid femur. Hind femur reaching apex of abdomen; hind tibia distinctly longer than hind femur. Tarsal formula 3-3-3; tarsomere III longest, subequal to length of tarsomeres I and II combined. Claws narrow, simple in shape. Fossula spongiosa absent in all legs.

Hemelytron (Figure 6) reaching or slightly surpassing apex of abdomen in midline; corium with hexagonal cubital cell; membrane with typical two cells; M strongly curved laterally at base of apical external cell; basal margin of apical internal cell convex.

Abdomen elongate oval, with simple lateral margin; dorsal laterotergites narrowly exposed in dorsal view; ventral surface with median longitudinal ridge running from base of sternite II to apex of sternite VII. Posterior margin of sternite VI widely concave anteriorly (Figure 2C and Figure 4C). Posterior margin of segment VII widely incised at midportion. Segment VIII clearly exposed in lateral and ventral views, with a widely concave posteromedian margin.

Male genitalia: Pygophore (Figure 7A–C and Figure 8A–C) oblong, with a wide, flattened, widely concave superoposterior margin; transverse bridge slender; median process weakly developed. Paramere (Figure 7D–F and Figure 8D–F) short, curved, covered with dense setae on apical two-thirds, with one subapical keel on inner surface, apically rounded. Phallus (Figure 7G–I and Figure 8G–I) relatively small; basal plate extension longer than basal plate arms; dorsal phallothecal sclerite well developed, with a round posterior margin; struts slender, parallel in majority of their lengths and fused at apices; ventral sclerite of phallosoma developed, narrow; endosoma elongate, largely membranous.

Macropterous female (Figure 2D–F and Figure 4D–F). **Vestiture.** Similar to that of male but differs in the following characteristics: antennal scape and basal half to three-fourths of pedicel only with tiny scale-like setae; flagellomeres and apical one-fourth to half of pedicel with short suberect setae and short, decumbent, whitish pubescence.

**Structure.** Similar to that of male but differs in the following characteristics: body robust, subfusiform (Figure 2D–F and Figure 4D–F); head (Figure 5C,D,G,H) thickened, with postocular region nearly parallel-sided or weakly convergent posteriorly; eye (Figure 5C,D,G,H) smaller, moderately protruding laterally, ventral margin reaching ventral head margin in lateral view, ventromedial margin far remote from each other; ocellus (Figure 5C,D,G,H) small, slightly elevated; antennal scape short, distinctly shorter than head, curved; length of pronotum slightly longer than width across humeral angles; anterior pronotal lobe slightly shorter than posterior lobe; humeral angles blunt, angulated or acute, weakly protruding laterally (Figure 5C,D,G,H); apical process of scutellum horizontal or slightly curved upwards (Figure 5C,D,G,H); hind femur not reaching apex of abdomen; hemelytron reaching anterior portion of tergite VI to posterior portion of tergite VIII in midline; abdomen subfusiform, with dorsal laterotergites widely exposed in dorsal view; posterior margin of sternite VI sharply incised anteriorly at midpoint (Figure 2F and Figure 4F).

Female genitalia: Tergite VIII (Figure 9A,B,D,E) short, transverse; tergite IX (Figure 9A,B,D,E) large, subtriangular, declined; valvifer I (Figure 9C,F) broad, with straight inner margin, apically blunt; valvula I (Figure 9C,F) short, apically blunt; valvula II (Figure 9B,C,E,F) subtriangular, with many peg-like setae on lateral surface.

**Diversity and distribution.***Argolis* currently contains 16 species, 14 of which are in the Afrotropical Realm and two in South and East Asia.

**Remarks.** In the past few decades, the genus was more commonly referred to as *Caunus* Stål, 1865, because *Argolis* Stål, 1861 was considered a junior homonym of the butterfly genus “*Argolis*” (Boisduval 1836: 2 [39]). However, Swanson [24] pointed out that the correct spelling for “*Argolis*” in Boisduval [39] was *Ergolis* Boisduval, 1836, and therefore *Argolis* Stål, 1861 is a valid name as a genus of Stenopodainae.

### 3.2. Key to the Asian Species of Argolis

**Figure 1:**
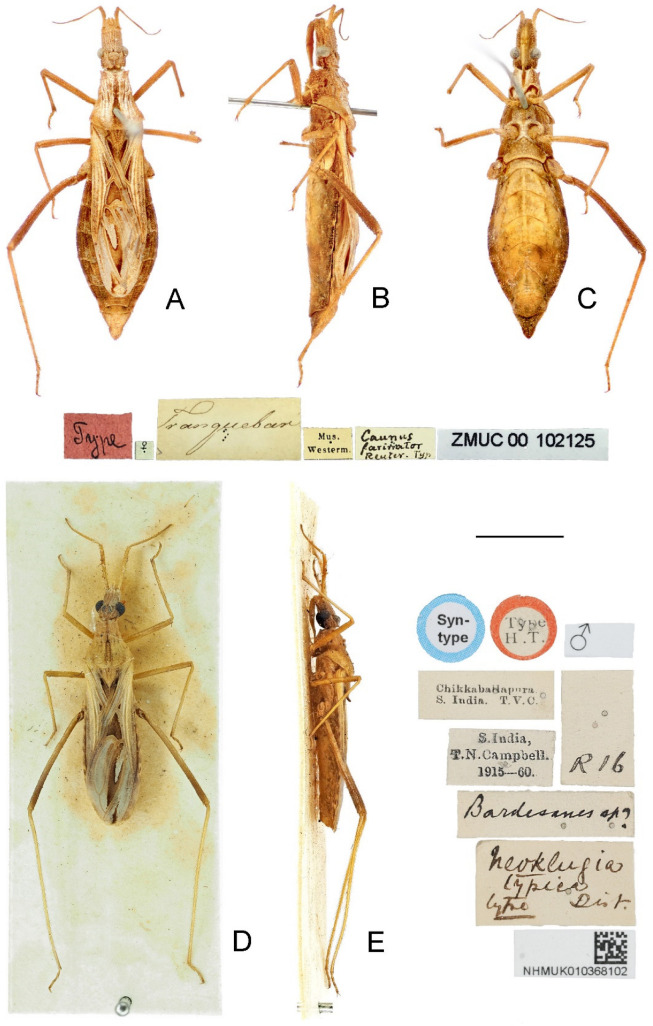
Name-bearing types of *Caunus farinator* Reuter, 1882 and *Neoklugia typica* Distant, 1919, habitus with labels: (**A**–**C**) *C. farinator*, lectotype, female; (**D**,**E**) *N. typica*, lectotype, male. (**A**,**D**) Dorsal; (**B**,**E**) lateral; (**C**) ventral. Scale bar: 5.0 mm. ©ZMUC (A–C) and ©BMNH (D,E).

**Figure 2:**
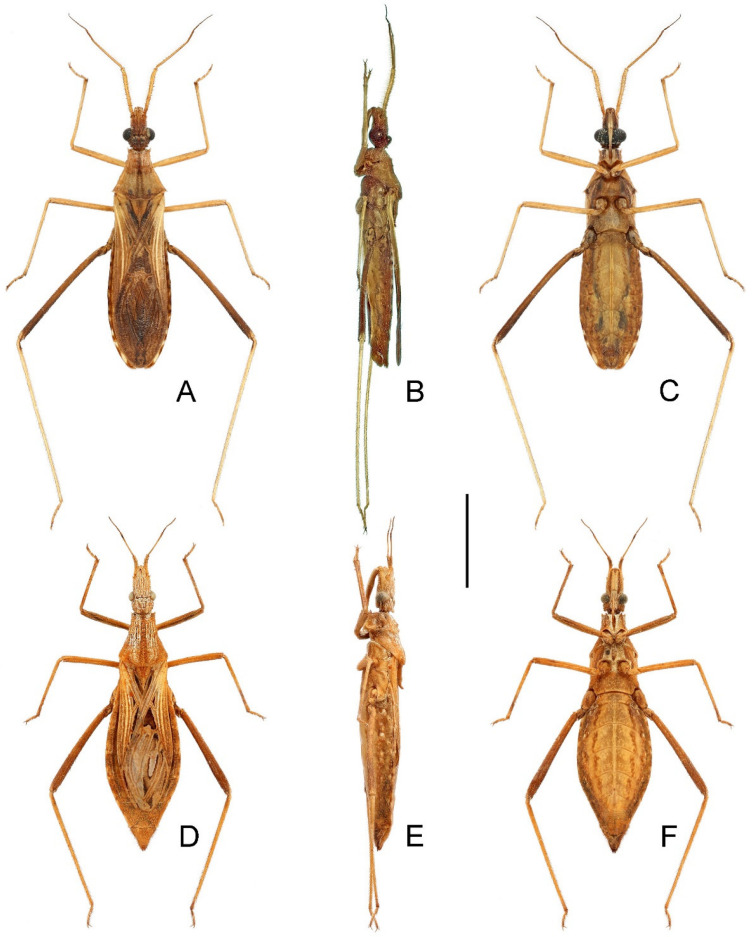
*Argolis farinator* (Reuter, 1882), habitus: (**A**–**C**) non-type male; (**D**–**F**) non-type female. (**A**,**D**) Dorsal; (**B**,**E**) lateral; (**C**,**F**) ventral. Scale bar: 5.0 mm.

1 Body generally yellowish brown (Figure 1 and Figure 2); lateral margin of pronotum with one granulation at midpoint (Figure 5I); tibiae yellowish brown (Figure 2); ventral surface of pygophore not emarginate subapically in lateral view (Figure 7B).

***A. farinator* (Reuter)**

**Figure 3:**
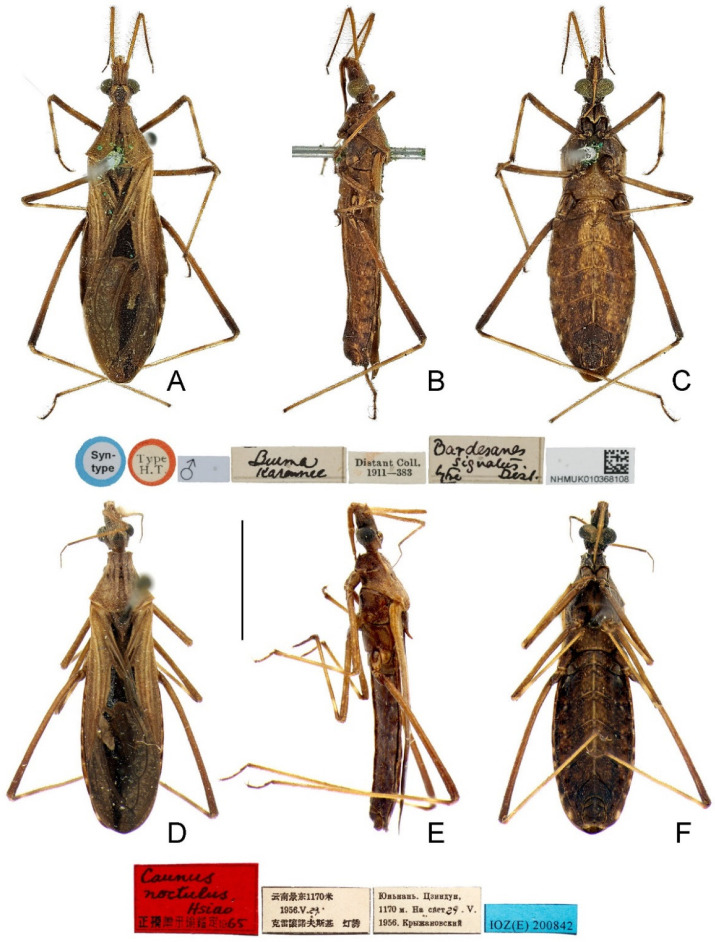
Name-bearing types of *Bardesanes signatus* Distant, 1909 and *Caunus noctulus* Hsiao, 1977, habitus with labels: (**A**–**C**) *B. signatus*, lectotype, male; (**D**–**F**) *C. noctulus*, holotype, male. (**A**,**D**) Dorsal; (**B**,**E**) lateral; (**C**,**F**) ventral. Scale bar: 5.0 mm. ©BMNH (Figure 3A–C) and ©IZAS (Figure 3D–F).

**Figure 4:**
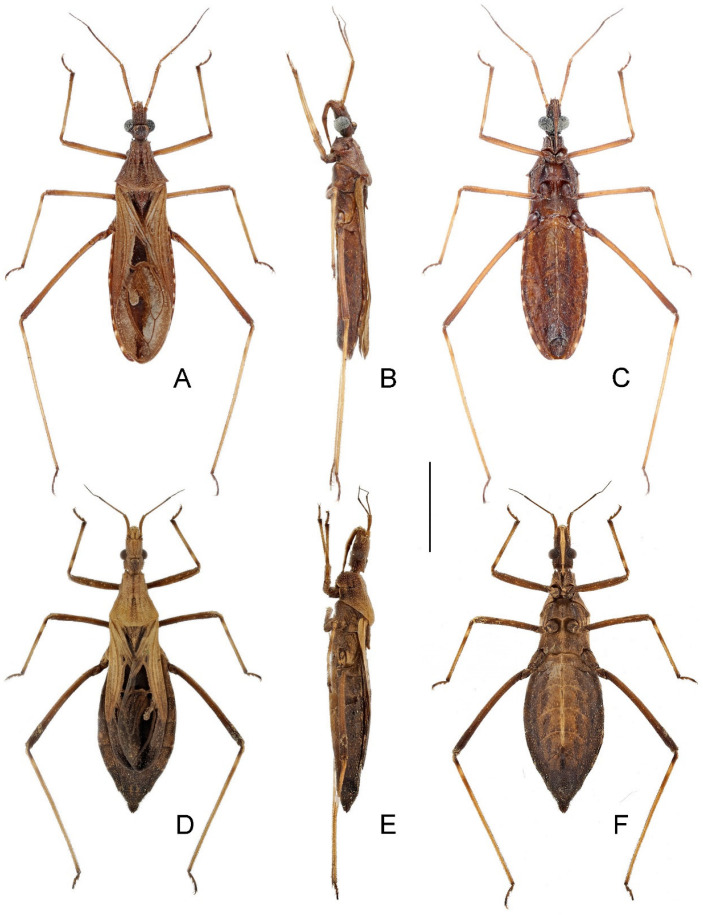
*Argolis signata* (Distant, 1909), **comb. nov.**, habitus: (**A**–**C**) non-type male; (**D**–**F**) non-type female. (**A**,**D**) Dorsal; (**B**,**E**) lateral; (**C**,**F**) ventral. Scale bar: 5.0 mm.

**Figure 5:**
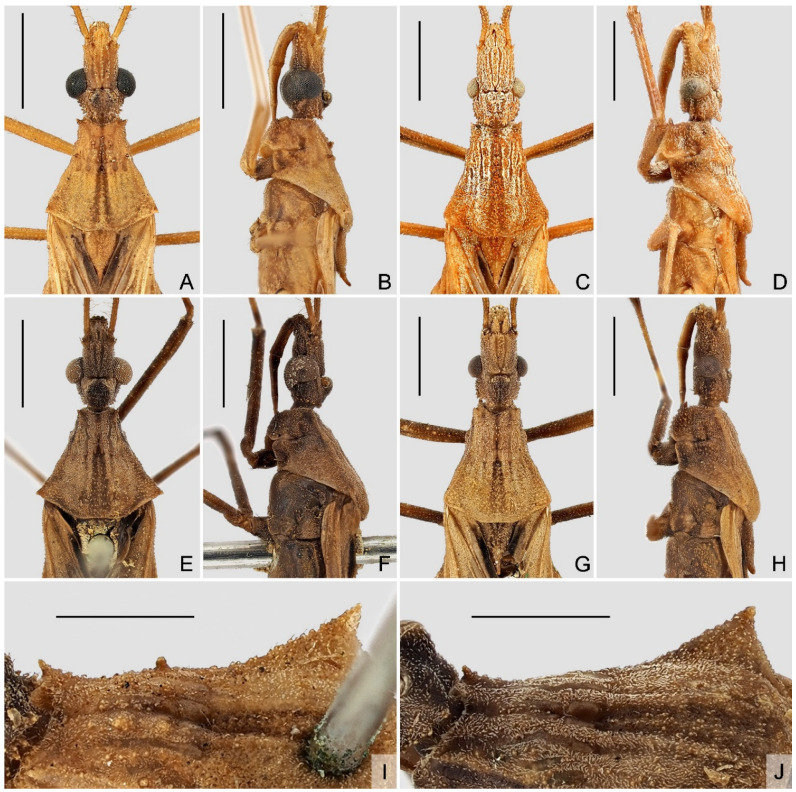
Morphological characters of two Asian *Argolis* species: (**A**–**D**,**I**) *A. farinator* (Reuter, 1882); (**E**–**H**,**J**) *A. signata* (Distant, 1909), **comb. nov.** (**A**–**H**) Anterior part of body; (**I**,**J**) lateral margin of pronotum. (**A**,**B**,**E**,**F**,**I**,**J**) Non-type male; (**C**,**D**,**G**,**H**) non-type female. (**A**,**C**,**E**,**G**) Dorsal; (**B**,**D**,**F**,**H**–**J**) lateral. Scale bar of (**A**–**H**) = 2.0 mm; of (**I**,**J**) = 1.0 mm.

- Body generally dark brown (Figure 3 and Figure 4); lateral margin of pronotum simple, lacking granulation (Figure 5J); tibiae yellowish brown, each with one basal and one subbasal dark brown annuli (Figure 4); ventral surface of pygophore strongly emarginate subapically in lateral view (Figure 8B).

***A. signata* (Distant), comb. nov.**

### 3.3. Argolis farinator (Reuter, 1882)

**Figure 6:**
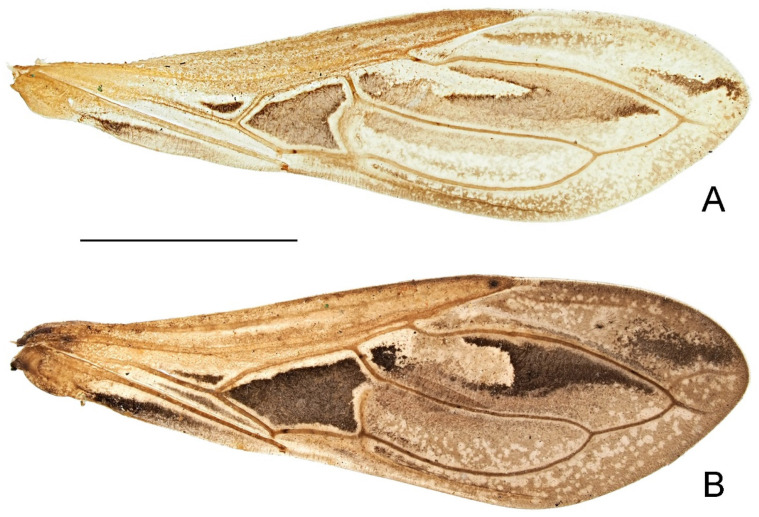
Hemelytron of two Asian *Argolis* species: (**A**) *A. farinator* (Reuter, 1882), non-type male; (**B**) *A. signata* (Distant, 1909), **comb. nov.**, non-type male. Scale bar of 2.5 mm.

**Figure 7:**
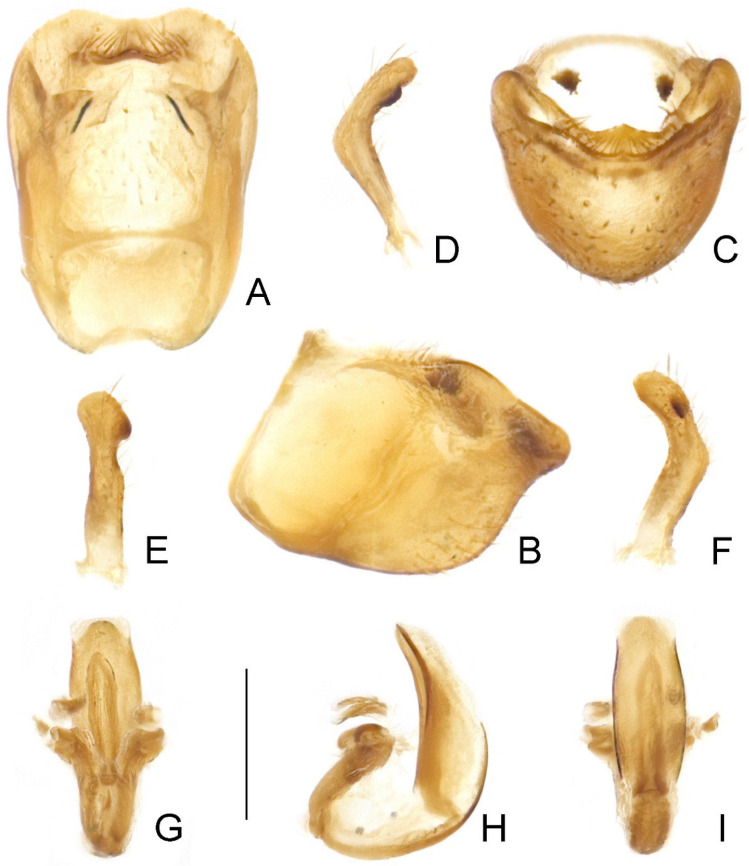
Male genitalia of *Argolis farinator* (Reuter, 1882): (**A**–**C**) pygophore; (**D**–**F**) paramere; (**G**–**I**) phallus. (**A**,**G**) Dorsal; (**B**,**H**) lateral; (**C**) caudal; (**I**) ventral. Scale bar: 0.5 mm.

(Figure 1, Figure 2, Figure 5, Figure 6, Figure 7 and Figure 9)

*Caunus farinator* Reuter, 1882: 752 [12] (protologue); Lethierry and Severin (1896: 89) [21] (catalogue, distribution); Distant (1903: 233) [26] (redescription, distribution, figure); Maldonado-Capriles (1990: 498) [1] (catalogue, distribution); Livingstone et al. (1998: 223) [40] (morphology of mandibular stylets, photo); Ambrose (2003: 97) [41] (listed, bionomics); Gupta et al. (2005: 130) [31] (catalogue, distribution); Ambrose (2006: 2405) [32] (listed, distribution); Thanasingh and Ambrose (2011: 43) [42] (listed, record). Syntype (♀): India, Tamil Nadu, Tharangambadi, ZMUC.

*Neoklugia typica* Distant, 1919: 72 [38] (protologue); Maldonado-Capriles (1990: 508) [1] (catalogue, distribution); Ambrose (2006: 2405) [32] (listed, distribution). Syntypes (2♂♂): India, Karnataka, Chikkaballapura, BMNH. **New subjective synonym.**

*Bardesanes sericenotatus* Livingstone and Ravichandran, 1989: 41 [43] (protologue); Livingstone et al. (1998: 223) [40] (morphology of mandibular stylets, photo); Ravichandran et al. (1998: 446) [44] (morphology of male genitalia, figure); Ambrose (2003: 97) [41] (listed, bionomics); Ambrose (2004: 443) [45] (listed); Gupta et al. (2005: 132) [31] (catalogue, distribution); Ambrose (2006: 2405) [32] (listed, distribution). Holotype (♂): India, Tamil Nadu, Vadavalli, BUCI. **New subjective synonym.**

*Argolis farinator*: Swanson (2018: 180) [24] (new combination).

*Bardesanes signatus* (non Distant, 1909): Hasegawa (1980: 37) [46] (record, photo); Chandra et al. (2013: 141) [47] (redescription, record, photo). Misidentification.

**Type material examined. *Caunus farinator *Reuter, 1882. Lectotype** (here designated) (♀): “Type” [hw., red rectangle]; “♀” [hw.]; “Tranquebar” [hw.]; “Mus.\Westerm.” [pr.]; “Caunus\farinator\Reuter. Typ” [hw.]; “ZMUC 00 102125” [pr.] (ZMUC).

***Neoklugia typica *Distant, 1919. Lectotype** (here designated) (♂): blue-margined syntype disc [pr.]; red-margined holotype disc [pr.]; “♂” [pr.]; “Chikkaballapura\S. India. T.V.C.” [pr.]; “S. India,\T.N.Campbell.\1915–60.” [pr.]; “R16” [hw.]; “Bardesanes sp?” [hw.]; “Neoklugia\typica\type Dist.” [hw.]; “NHMUK010368102” [pr.] (BMNH). **Paralectotype**: blue-margined syntype disc [pr.]; “♂” [pr.]; “Chikkaballapura\S. India.\T. V. Campbell.” [pr.]; “S. India,\T.N.Campbell.\1915–60.” [pr.]; “R16” [hw.]; “NHMUK 013587630” [pr.] (1♂, BMNH).

**Additional materials examined. INDIA**. **Karnataka**: Chikkaballapura, leg. T.V. Campbell (5♂2♀, BMNH). **Maharashtra**: Mumbai, leg. Leith (1♀, BMNH). **Rajasthan**: Jhunjhunu, Pilani, leg. R. Kumar (1♂, BMNH). **Tamil Nadu**: Erode, Hasanur, 29.iv.1937 (1♂, BMNH). **Uttarakhand**: Almora, Ranikhet, leg. G.C. Champion (1♂, BMNH). **Exact locality unknown**: leg. W.W. Saunders (1♀, BMNH). **PAKISTAN**. **Sindh**: Karachi, Malir, 10.ix.1959, leg. A. Habib, on ground (1♀, BMNH); same locality and collector as preceding, 20.x.1960, at light (1♂1♀, BMNH). **SRI LANKA**. **North Western**: Puttalam, leg. E.E. Green (1♂, BMNH).

**Diagnosis.** Body generally yellowish brown (Figure 1 and Figure 2); head and prothorax with prominent setigerous tubercles (Figure 5A–D); anteocular region more than 2.7 times as long as postocular; lateral margin of pronotum with one small granulation at midpoint (Figure 5I); tibiae uniformly yellowish brown (Figure 2); cubital cell of hemelytron shorter than half of length of apical external cell (Figure 6A); pygophore weakly expanded posteriorly in dorsal view (Figure 7A), ventral surface not emarginate subapically in lateral view (Figure 7B); paramere slender, with short subapical keel (Figure 7D–F).

**Redescription.** Macropterous male (Figure 2A–C). **Coloration.** Generally yellowish brown. Head with ocellar tubercle and eye blackish brown; ocellus, oblique stripe outside of ocellus and ventral surface of head brown to dark brown. Antennal flagellomeres and apex of pedicel dark brown. Labium with lateral surface brown and ventral surface yellow. Pronotum with dark brown median longitudinal stripe nearly throughout its length, stripe narrow on anterior lobe, wide and separated on posterior lobe (Figure 5A); lateral margin and apical process of scutellum slightly darkened; thoracic sterna and ventral third of pleura pale brown to brown. Legs unicolored; hind femur dark brown, with apical third slightly darkened (Figure 2A–C); inner surface of base of hind tibia dark brown (Figure 2A,C). Hemelytron pale greyish brown, with basal portion, exocorium and veins pale yellowish brown to yellowish brown (Figure 6A); corium with large blackish patch almost filled cubital cell, and small, oblique triangular, blackish patch at base of cubital cell (Figure 6A); clavus with blackish stripe along medial border, gradually becoming paler towards apex (Figure 6A); membrane with elongate pale brown patch along outer border of apical internal cell, elongate whitish patch surrounded by large, irregular, dark brown patch in apical external cell, and small, elongate, blackish patch at apex (Figure 6A). Basal, submedian and apical spots of each dorsal laterotergite yellow; ventral surface of abdomen with one pair of disrupted, obscure, brown lateral stripes; outer third of both sides of sternites mottled with pale brown suffusion.

**Vestiture.** As in the redescription of the genus. Setigerous tubercles on head and prothorax prominent.

**Structure.** Head (Figure 5A,B) 1.3 times as long as width across eyes, width across eyes 2.2 times as broad as interocular space; anteocular region 2.7 times as long as postocular; mandibular plates slightly divergent anteriorly, slightly curved upwards; postocular region with paired prominent tubercles on posterior head margin. Antennal scape 1.1 times as long as head; pedicel 1.1 times as long as scape. Pronotum (Figure 5A,B) with length along midline 0.9 times as long as width across humeral angles; anterolateral angles acute, projecting anterolaterally; anterior lobe with four distinct granulations on disc, and one pair of small granulations laterally before transverse sulcus (Figure 5I); humeral angles distinctly protruding posterolaterally (Figure 5I). Cubital cell of hemelytron relatively small, shorter than half of length of apical external cell (Figure 6A). Abdomen 2.15 times as long as its maximum width.

Male genitalia: Pygophore (Figure 7A–C) finely expanded posteriorly in dorsal view; ventral surface not emarginate subapically in lateral view; median process short, wide, subtriangular. Paramere (Figure 7D–F) relatively slender, slightly constricted beyond midpoint; subapical keel relatively short. Phallus as shown in Figure 7G–I; basal plate arms of articulatory apparatus gradually divergent apically, enclosing an elongate basal foramen (Figure 7G); phallosoma elongate oval, with ventral sclerite in basal half (Figure 7H); struts simply curved at bases (Figure 7I).

Macropterous female (Figure 2D–F). **Coloration.** Similar to that of male. Dorsal laterotergites of abdomen largely dark brown, with indistinct yellowish suffusion posterolaterally; tergite VIII with one pair of blackish spots in anterior half (Figure 9A).

**Vestiture.** As in the redescription of the genus. Short suberect setae occupying apical half of antennal pedicel.

**Structure.** Similar to that of male but differs in the following characteristics: head (Figure 5C,D) 1.7 times as long as width across eyes, width across eyes 1.75 times as broad as interocular space; anteocular region 2.8 times as long as postocular; mandibular plates nearly parallel, horizontal. Antennal scape 0.4 times as long as head; pedicel 1.45 times as long as scape. Humeral angles of pronotum angulated (Figure 5C). Apical process of scutellum slightly curved upwards (Figure 5D). Abdomen 2.1 times as long as its maximum width.

Female genitalia: Apical half of tergite IX relatively broad in dorsal view (Figure 9A); valvula II relatively longer, with sharp apex (Figure 9B,C).

**Measurements** [in mm, ♂ (n = 12)/♀ (n = 7)]. Length of body: to apex of hemelytron 13.20–15.40/-; to apex of abdomen 13.00–15.00/15.50–15.80; length of head 2.10–2.40/2.50; length of anteocular region 1.10/1.30; length of postocular region 0.30–0.50/0.50; width across eyes 1.60–1.90/1.50–1.55; interocular space 0.70–0.90/0.90–1.00; interocellar space 0.40/0.40–0.45; length of antennal segments I–IV = 2.10–2.90/1.10, 2.20–3.10/1.50, 0.35–0.40/0.30–0.35, 0.90–1.00/0.75–0.90; length of visible labial segments I–III = 1.10–1.30/1.10, 0.85–1.10/1.10, 0.60–0.80/0.65–0.70; length of pronotum 2.40–3.00/2.70–2.90; length of anterior pronotal lobe 1.10–1.40/1.30–1.40; length of posterior pronotal lobe 1.30–1.70/1.40–1.50; width of anterior pronotal lobe 1.40–1.60/1.50–1.60; width of posterior pronotal lobe 2.70–3.30/2.60–2.70; median length of scutellum 1.25–1.40/1.30–1.40; basal width of scutellum 0.90–1.10/1.00; length of fore femur, tibia, tarsus = 3.00–3.50/2.90–3.10, 3.20–3.90/2.90–3.10, 0.90–1.05/0.80–0.90; length of mid femur, tibia, tarsus = 3.20–4.00/3.00–3.10, 3.65–4.40/3.50, 0.90–1.05/0.85–0.90; length of hind femur, tibia, tarsus = 5.60–6.40/5.30–5.60, 8.00–8.70/6.50–7.20, 1.20–1.35/1.00–1.20; length of hemelytron 9.00–10.70/8.30; length of abdomen 7.10–8.20/8.90–9.00; maximum width of abdomen 2.50–3.90/4.10–4.50.

**Figure 10:**
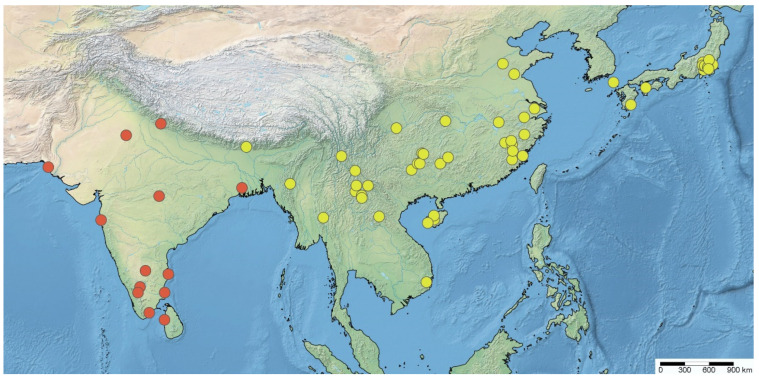
Known distribution of *Argolis* species from Asia. Red circle = *A. farinator* (Reuter, 1882); yellow circle = *A. signata* (Distant, 1909), **comb. nov.**

**Distribution. INDIA**–**Karnataka**: Chikkaballapura*; **Kerala** [31]; **Madhya Pradesh**: Chhindwara [47]; **Maharashtra**: Mumbai*; **Rajasthan**: Jhunjhunu*; **Tamil Nadu**: Chennai [46], Erode*, Mayiladuthurai*, Thoothukudi [42], Vadavalli [43]; **West Bengal**: Kolkata [31]; **Uttarakhand**: Almora*. **PAKISTAN**–**Sindh**: Malir*. **SRI LANKA**–**North Western**: Puttalam* (Figure 10).

**Bionomics.** Habitat recorded from India is agroecosystem [41,43] (as *B. sericenotatus* and *C. farinator*). It may be a ground-dwelling species that has been found on the ground (present study) or under boulders [41]. This species is also attracted to light [[41]; present study].

**Remarks.** Reuter [12] described *Caunus farinator* based on an unspecified number of female specimens (syntypic) from “Tranquebar” (=Tharangambadi; Tamil Nadu, India). The type depository provided in the original description is “Mus. Havn. in Coll. Westermanni”, which refers to Westermann’s collection in ZMUC. One female specimen (Figure 1A–C), matching the original collection data and bearing a red type label and an O.M. Reuter’s handwritten identification label, was found in the collection of ZMUC. This specimen fits the original description and therefore is a syntype of *C. farinator*, and it is here designated as the lectotype of this species.

*Neoklugia typica* was described based on an unspecified number and sex of specimens (syntypic) from “S. India; Chikkaballapura” (=Chikkaballapura; Karnataka, India) [38]. Nine specimens (seven males and two females) preserved in the collection of the BMNH match the original collection data. Since *Neoklugia* was apparently described from males as Distant [38] stated that the “first joint of the antennae [was] about as long as the pronotum, distinctly finely hirsute”, the females cannot be types. Of the seven males, two have the registration number “1915-60”, two have “1926-171” and the other three have “1930-599”. The registration numbers indicate when the specimens were acquired by BMNH, and any specimen obtained after the original description may or may not be a type [48,49]. Therefore, only the two males with the registration number “1915-60” are considered syntypes of *N. typica*. We here select the specimen bearing a red holotype disc and a W.L. Distant’s handwritten identification label (Figure 1D,E) as the lectotype of this species. Examination of the female specimens collected together with the males recovered sexual dimorphism in this species, which is a common phenomenon in *Argolis* (see discussion below). Based on the similarity of females to the female type of *A. farinator* we concluded that the species are conspecific. Therefore, we propose the following new subjective synonymy: *Argolis farinator* (Reuter, 1882) = *Neoklugia typica* Distant, 1919, **syn. nov.**

*Bardesanes sericenotatus* was described based on a male holotype and two paratypes (one male and one female) from Vadavalli, Tamil Nadu, India [43]. A dorsal habitus illustration of the male was provided along with the original description, and the phallus of this species was illustrated by Ravichandran et al. [44]. Although the type material of this species deposited in BUCI was not examined during this study, several morphological characters documented in Livingstone & Ravichandran [43] and Ravichandran et al. [44] indicate the species agrees perfectly with *A. farinator* in body size and coloration, as well as the following morphological characters: “postocular area with a tubercular process behind each ocellus, porrectly pointing backward[s] from vertex”; “anterior lobe [of pronotum] with a pair of minute discal tubercles on either side of the median foveation”; “two pairs of discal small tubercles in the anterior lobe of pronotum”. As these two species could not be distinguished by morphological characters, and also due to the proximality of their type localities, the following new subjective synonymy is proposed: *Argolis farinator* (Reuter, 1882) = *Bardesanes sericenotatus* Livingstone & Ravichandran, 1989, **syn. nov.**

### 3.4. Argolis signata (Distant, 1909), **comb. nov.**

**Figure 8:**
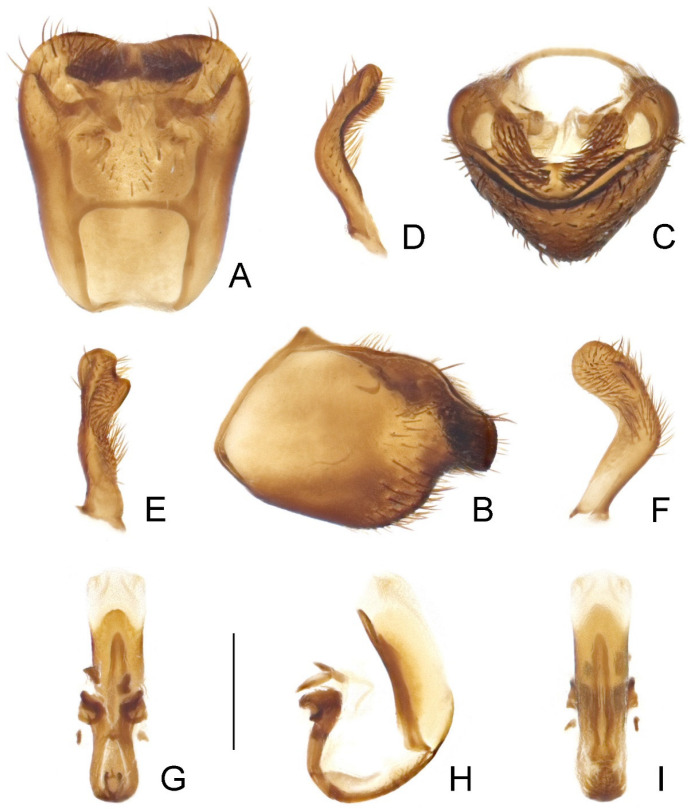
Male genitalia of *Argolis signata* (Distant, 1909), **comb. nov.**: (**A**–**C**) pygophore; (**D**–**F**) paramere; (**G**–**I**) phallus. (**A**,**G**) Dorsal; (**B**,**H**) lateral; (**C**) caudal; (**I**) ventral. Scale bar: 0.5 mm.

(Figure 3, Figure 4, Figure 5, Figure 6 and Figure 8)

*Bardesanes signatus* Distant, 1909: 364 [36] (protologue); Distant (1910: 188) [37] (redescription, distribution, figure); George (1988: 212) [50] (morphology of female genitalia, figure); Maldonado-Capriles (1990: 495) [1] (catalogue, distribution). Syntype (♂): Myanmar, Kayah, BMNH.

*Caunus noctulus* Hsiao, 1977: 74, 81 [27] (protologue); Hsiao and Ren (1981: 477) [28] (redescription, distribution, figure, photo); Li (1990: 27) [51] (redescription, distribution, figure); Maldonado-Capriles (1990: 498) [1] (catalogue, distribution); Ren (1992: 80) [52] (morphology of egg, bionomics, photo); Tian (1993: 186) [53] (redescription, distribution, bionomics, figure); Chen et al. (1995: 211) [54] (record); Putshkov and Putshkov (1996: 209) [30] (catalogue, distribution); Ren et al. (1999: 174) [55] (redescription, distribution, bionomics); Ding et al. (2000: 167) [56] (listed); Hua (2000: 207) [57] (listed, distribution); Liu and Chen (2003: 216) [58] (listed); Liu et al. (2003: 198) [59] (listed); Ishikawa et al. (2005: 264) [60] (diagnosis, distribution, record, photo); Yuan et al. (2006: 56) [61] (listed, distribution); Cui et al. (2007: 169) [62] (type material); Zhao and Cai (2007: 199) [63] (redescription, distribution); Ye (2009: 57) [64] (listed, distribution); Cao et al. (2011: 51) [6] (redescription, distribution, record, figure); Li (2011: 140) [65] (listed, distribution); Ishikawa and Miyamoto (2012: 277) [33] (diagnosis, distribution, record, photo); Nozawa (2012: 27) [66] (diagnosis, record); Okuda (2012: 37) [67] (record, bionomics); Ishikawa (2016: 449) [35] (catalogue, distribution); Okuda and Uchida (2019: 62) [68] (record, bionomics, photo); Abe and Okuda (2020: 47) [69] (record, bionomics, photo); Okuda (2020a: 45) [70] (record, photo); Okuda (2020b: 5) [71] (photo). Holotype (♂): China, Yunnan, Jingdong, IZAS. **New subjective synonym.**

*Caunus* sp.: Nozawa (1978: 371) [72] (diagnosis, record, bionomics).

*Bardesanes* sp.: Hasegawa (1980: 36) [46] (record, bionomics, photo); Nozawa (1990: 7) [73] (diagnosis, record, bionomics, figure).

*Argolis noctula*: Swanson (2018: 180) [24] (new combination).

**Type material examined. *Bardesanes signatus *Distant, 1909. Lectotype** (here designated) (♂): blue-margined syntype disc [pr.]; red-margined holotype disc [pr.]; “♂” [pr.]; “Burma\Karennee” [hw.]; “Distant Coll.\1911–383” [pr.]; “Bardesanes\signatus.\type Dist.” [hw.]; “NHMUK010368108” [pr.] (BMNH).

***Caunus noctulus* Hsiao, 1977. Holotype** (♂): “云南景东1170米 [‘Yunnan Jingdong 1170 m’, pr.]\1956.V. [pr.] 29 [hw.]. [pr.]\克雷讓諾夫斯基 灯誘 [‘Kryzhanovsky light trap’, pr.]”; “Юньнань. Цзиндун, [‘Yunnan. Jingdong,’, pr.]\1170 м. На свет [‘1170 m. To light’, pr.] 29 [hw.]. V. [pr.]\1956. Крыжанoвскнй [‘1956. Kryzhanovsky’, pr.]”; “Caunus [hw.]\noctulus [hw.]\Hsiao [hw.]\正模萧采瑜鑑定19 [‘holotype identified by Hsiao Tsai-Yu 19′, pr.] 65 [hw.]” [red rectangle]; “IOZ(E) 200842” [pr., in blue rectangle] (IZAS). **Paratypes**: “云南景东1170米 [‘Yunnan Jingdong 1170 m’, pr.]\1956. [pr.] V [hw.]. [pr] 29 [hw.]. [pr.]\扎古良也夫 灯誘 [‘Zagulyaev light trap’, pr.]”; “Юньнань. Цзиндун, [‘Yunnan. Jingdong,’, pr.]\1170 м. На свет [‘1170 m. To light’, pr.] 29 [hw.]. [pr.] V [hw.]. [pr.]\1956. Загуляев [‘1956. Zagulyaev’, pr.]”; “ALLOTYPE” [pr., green rectangle]; “Caunus [hw.]\noctulus [hw.]\Hsiao [hw.]\配模萧采瑜鑑定19 [‘allotype identified by Hsiao Tsai-Yu 19′, pr.] 65 [hw.]” [red rectangle]; “IOZ(E) 200843” [pr., in blue rectangle] (1♀, IZAS). “云南景东董家坟1250米 [‘Yunnan Jingdong Dongjiafen 1250 m’, pr.]\1956.VI. [pr.] 8 [hw.]. [pr.]\克雷讓諾夫斯基 [‘Kryzhanovsky’, pr.]”; “Юньнань. 10 км. N Цзин- [‘Yunnan. 10 km N Jing-’, pr.]\дуна, 1250 м. [‘dong, 1250 m’, pr.] 8 [hw.]. VI. 1956. [pr.]\Крыжанoвскнй [‘Kryzhanovsky’, pr.]”; “PARATYPE” [pr., yellow rectangle]; “IOZ(E) 200844” [pr., in blue rectangle] (1♂, IZAS). “云南景东董家坟1250米 [‘Yunnan Jingdong Dongjiafen 1250 m’, pr.]\1956. [pr.] V [hw.]. [pr.] 30 [hw.]. [pr.]\扎古良也夫 [‘Zagulyaev’, pr.]”; “Юньнань. 10 км. И Цзин- [‘Yunnan. 10 km N Jing-’, pr.]\дуна, 1250 м. [‘dong, 1250 m’, pr.] 30 [hw.]. [pr.] V [hw.]. 1956. [pr.]\Загуляев [‘Zagulyaev’, pr.]”; “PARATYPE” [pr., yellow rectangle]; “IOZ(E) 200845” [pr., in blue rectangle] (1♀, IZAS).

**Additional materials examined. CHINA**. **Fujian**: Quanzhou, Dehua, Jiuxianshan, 1400 m, 29.vi.2014, leg. Yi-Ting Chung (1♀, CAU); Fuzhou, Gulou, West Lake Park, 30.v.1960, leg. Shumin Fang (1♂, CAU); Nanping, Wuyishan, Xiaowuyi, 30.vi.1979, leg. Chikun Yang (1♂, CAU). **Guizhou**: Qiandongnan, Rongjiang, Pingyang, 920–970 m, 3.vi.2005, leg. Ping Zhao, by light trap (1♂, CAU); Qiannan, Libo, Maolan, 11.vi.2005, leg. Ping Zhao (1♀, CAU); Qiannan, Libo, Xiaoqikong, 17.v.1999, leg. Qiongzhang Song (1♀, CAU). **Hainan**: Baisha, Yuanmen, Hongxin vill., 10.x.2008, leg. Wenjie Zhang (1♂, CAU); Danzhou, Nada, Tropical Botanical Garden, 8.v.2007, leg. Wenjie Zhang (1♂, CAU); Ledong, Jianfengling, Chahekou, 220 m, 5.v.2007, leg. Hongbin Liang, by sweep net (1♂, IZAS). **Jiangsu**: Suzhou, leg. Chenfu Wu (1♀, BMNH). **Shandong**: Taian, Taishan, 18.vii.1986, leg. Qiang Li (1♂, CAU). **Yunnan**: Dali, Yunlong, Jiancao, Longmashan, 2954 m, 21.vi.2020, leg. Xinjie Zhao (1♂, CAU); Honghe, Lvchun, Huanglianshan, Qimaba, 10.v.2012 (1♀, CAU); Puer, Simao, 26.v.1984, leg. Yousheng Zhou (1♂, CAU); Xishuangbanna, Jinghong, Mengyang, 800 m, 27.v.2006, leg. Hesheng Wang (1♂1♀, CAU); Xishuangbanna, Mengla, 650 m, 9.vi.1991, leg. Wanzhi Cai (1♀, CAU); Xishuangbanna, Mengla, Yaoqu, 620 m, 11.v.1991, leg. Wanzhi Cai, by light trap (1♂, CAU). **Zhejiang**: Hangzhou, Lin’an, Xitianmushan, 20.vi.1984, leg. Xue Li (1♀, CAU). **Exact locality unknown** (1♀, CAU). **INDIA**. **West Bengal**: Pedong, 1897, leg. R. Oberthür (1♂1♀, MNHN). **JAPAN**. **Gumma**: Maebashi, Egi, Gumma Station, 23.vi.1969, leg. E. Hara, by light trap (1♂, NIAES). **Kanagawa**: Sagamihara, 25.vi.1960, leg. H. Takenaka (1♂, NIAES). **Tokyo**: exact locality unknown, 20.vi.1954, leg. T. Okazaki (1♀, NIAES). **LAOS**. **Xiangkhouang**: exact locality unknown, 25.iv.1919, leg. R.V. de Salvaza (1♂, BMNH); exact locality unknown, same collector as preceding, 5.v.1919 (1♀, BMNH); exact locality unknown, same collector as preceding, 8.v.1919 (1♂, BMNH). **MYANMAR**. **Chin**: Falam, Chin Hill, 17.vii.2019, leg. P.N. Kyaw (1♂, CAU). **VIETNAM**. **Lam Dong**: Lac Duong, Bidoup Nui Ba, Giang Ly Ranger Station, 1455 m, 11.v.2012, leg. Jianyun Wang (1♂, CAU).

**Diagnosis.** Body generally dark brown (Figure 3 and Figure 4); head and prothorax with minute setigerous tubercles (Figure 5E–H); anteocular region less than 2.2 times as long as postocular; lateral margin of pronotum simple, lacking small granulation (Figure 5J); tibiae yellowish brown, each with one basal and one subbasal dark brown annuli (Figure 4); cubital cell of hemelytron longer than half of length of apical external cell (Figure 6B); pygophore distinctly expanded posteriorly in dorsal view (Figure 8A), ventral surface strongly emarginate subapically in lateral view (Figure 8B); paramere stout, with wide subapical keel enclosing an arc with apex of paramere (Figure 8D–F).

**Redescription.** Macropterous male (Figure 4A–C). **Coloration.** Generally dark brown. Head with middle of postocular region (Figure 5E), eye and ventral surface blackish brown; ocellus brown. Antennal scape and basal two-thirds of pedicel yellowish brown, flagellomeres and apical third of pedicel dark brown. Labium with lateral surface brown and ventral surface yellow. Pronotum with indistinct, blackish, medial longitudinal stripe nearly throughout its length, stripe narrow on anterior lobe, wide and divergent on posterior lobe (Figure 5E); scutellum slightly darkened on disc, with apical process slightly paler; thoracic pleura and sterna with indistinct irregular darkened suffusion. Femora brown, each with base and apical third dark brown (Figure 4A–C); tibiae yellowish brown, each with base, narrow subbasal annulus and apex dark brown (Figure 4A–C). Hemelytron with coriaceous proximal portion brown and membrane dark greyish brown (Figure 6B); corium with large blackish patch almost filled cubital cell, and small, oblique triangular, blackish patch and narrow, oblique, blackish stripe at base of cubital cell (Figure 6B); clavus with blackish stripe along medial border, slightly becoming paler towards apex (Figure 6B); membrane with elongate whitish patch surrounded by large, irregular, blackish patch in apical external cell, and small, elongate, blackish patch at apex (Figure 6B). Basal, submedian and apical spots of each dorsal laterotergite yellow; ventral surface of abdomen yellowish brown at middle, with one pair of disrupted, obscure, blackish lateral stripes; sternite VII, segment VIII and pygophore slightly darkened.

**Vestiture.** As in the redescription of the genus. Setigerous tubercles on head and prothorax minute.

**Structure.** Head (Figure 5E,F) 1.2 times as long as width across eyes, width across eyes 2.35 times as broad as interocular space; anteocular region 2.2 times as long as postocular; mandibular plates distinctly divergent anteriorly, slightly curved upwards; postocular region with paired blunt tubercles on posterior head margin. Antennal scape 1.2 times as long as head; pedicel 1.15 times as long as scape. Pronotum (Figure 5E,F) with length along midline 0.9 times as long as width across humeral angles; anterolateral angles blunt to angulated; anterior lobe with four granulations on disc, posterior pair indistinct; humeral angles distinctly protruding laterally (Figure 5J). Cubital cell of hemelytron relatively large, longer than half of length of apical external cell (Figure 6B). Abdomen 2.1 times as long as its maximum width.

Male genitalia: Pygophore (Figure 8A–C) distinctly expanded posteriorly in dorsal view; ventral surface strongly emarginate subapically in lateral view; median process indistinct. Paramere (Figure 8D–F) relatively stout, slightly widened in apical half; subapical keel relatively wide, enclosing an arc with apex of paramere. Phallus as shown in Figure 8G–I; basal plate arms of articulatory apparatus widely separated at base, gradually convergent apically, enclosing a triangular basal foramen (Figure 8G); phallosoma elongate oblong, with ventral sclerite in basal third (Figure 8H); struts bisinuate at bases (Figure 8I).

**Figure 9:**
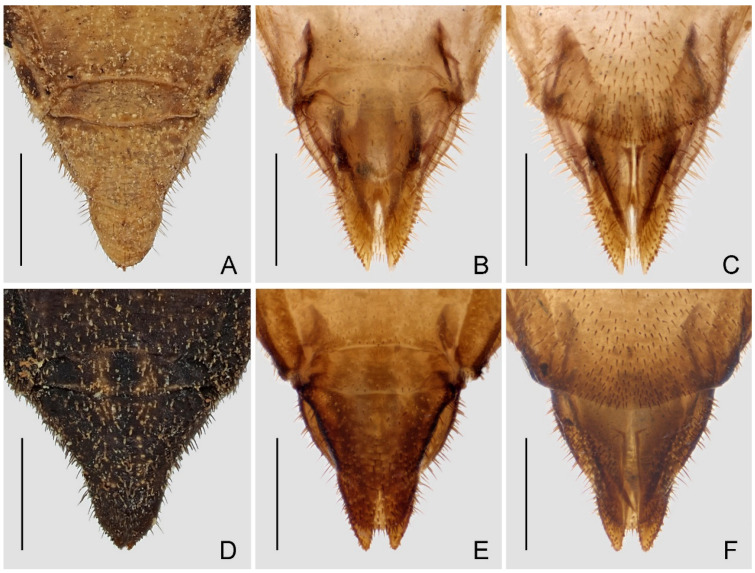
Female genitalia of two Asian *Argolis* species: (**A**–**C**) *A. farinator* (Reuter, 1882); (**D**–**F**) *A. signata* (Distant, 1909), **comb. nov.** (**A**,**B**,**D**,**E**) Dorsal; (**C**,**F**) ventral. Scale bar: 1.0 mm.

Macropterous female (Figure 4D–F). **Coloration.** Similar to that of male. Dorsal laterotergites of abdomen largely dark brown, with indistinct light-colored spots; tergite VIII with one pair of faint yellowish brown spots extending to anterior portion of tergite IX (Figure 9D).

**Vestiture.** As in the redescription of the genus. Short suberect setae occupying apical one-fourth of antennal pedicel.

**Structure.** Similar to that of male but differs in the following characteristics: head (Figure 5G,H) 1.6 times as long as width across eyes, width across eyes 1.7 times as broad as interocular space; anteocular region 2.1 times as long as postocular; mandibular plates slightly divergent anteriorly, horizontal. Antennal scape 0.5 times as long as head; pedicel 1.6 times as long as scape. Humeral angles of pronotum blunt (Figure 5G). Apical process of scutellum blunt, horizontal (Figure 5H). Abdomen 2.15 times as long as its maximum width.

Female genitalia: Apical half of tergite IX relatively narrow in dorsal view (Figure 9C); valvula II relatively shorter, with blunt apex (Figure 9E,F).

**Measurements** [in mm, ♂ (n = 20)/♀ (n = 13)]. Length of body: to apex of hemelytron 13.40–16.40/-; to apex of abdomen 13.10–16.50/15.70–17.70; length of head 2.00–2.70/2.50–2.80; length of anteocular region 1.00–1.40/1.30–1.40; length of postocular region 0.50–0.60/0.60–0.70; width across eyes 1.65–1.90/1.55–1.70; interocular space 0.70–0.90/0.90–1.00; interocellar space 0.30–0.40/0.35–0.40; length of antennal segments I–IV = 2.50–3.30/1.20–1.30, 2.90–3.60/1.85–2.10, 0.40–0.45/0.30–0.35, 0.75–1.00/0.75–0.80; length of visible labial segments I–III = 0.90–1.20/1.10–1.20, 0.95–1.25/1.15–1.25, 0.65–0.80/0.70–0.80; length of pronotum 2.50–3.10/2.70–3.00; length of anterior pronotal lobe 1.10–1.50/1.30–1.50; length of posterior pronotal lobe 1.40–1.60/1.40–1.50; width of anterior pronotal lobe 1.40–1.60/1.60–1.70; width of posterior pronotal lobe 2.70–3.30/2.90–3.10; median length of scutellum 1.10–1.50/1.30–1.50; basal width of scutellum 0.90–1.10/1.10–1.15; length of fore femur, tibia, tarsus = 3.50–4.40/3.40–3.60, 3.60–4.70/3.40–3.60, 0.90–1.10/0.90; length of mid femur, tibia, tarsus = 3.60–4.70/3.70–4.00, 3.90–5.20/4.00–4.10, 0.90–1.10/0.80–0.90; length of hind femur, tibia, tarsus = 5.90–8.20/6.40–6.50, 8.20–11.25/8.20–8.40, 1.20–1.50/1.15–1.20; length of hemelytron 9.20–11.20/9.00–9.40; length of abdomen 7.00–8.70/9.05–10.00; maximum width of abdomen 3.50–3.60/4.10–4.80.

**Distribution. CHINA**–**Fujian**: Dehua, Fuzhou, Guangze [55], Jianyang [28], Wuyishan, Yanping [58]; **Guangxi**: Fengshan, Lingui, Quanzhou [51]; **Guizhou**: Leishan [61], Libo, Rongjiang; **Hainan**: Baisha, Danzhou, Ledong*; **Henan** [6]; **Hunan**: Shimen [6]; **Jiangsu**: Suzhou*; **Jiangxi**: Pengze [56]; **Shandong**: Linyi [53], Taishan; **Sichuan**: Lu County [28]; **Yunnan**: Jingdong, Jinghong, Lvchun*, Mengla, Simao*, Yunlong*; **Zhejiang**: Lin’an*, Songyang [54]. **INDIA**–**West Bengal**: Pedong*. **JAPAN**–**Honshu**: Gunma, Ibaraki [69], Kanagawa [46], Saitama [72], Tochigi [35], Tokyo*; **Kyushu**: Miyazaki [67]; **Shikoku**: Ehime [35]; **Tsushima Is.**: Izuhara [46]. **KOREAN PENINSULA** [35]. **LAOS**–Xiangkhouang*. **MYANMAR**–**Chin**: Falam*; **Kayah**. **VIETNAM**. **Lam Dong**: Lac Duong* (Figure 10).

**Bionomics.** Published bionomic records of this species are from China and Japan. Its habitat was speculated to be wetlands near paddy fields [46,67], but most specimens were collected under lights [[46,52,68]; present study]. Ren [52] recorded that this species inhabits the rhizosphere of plants or on the ground, and the females lay their eggs in soil crevices, near soil clumps or under loose soil. It is probably a generalist predator, Tian [53] recorded lepidopteran caterpillars as its preys in Shandong, China. The surface structure of its eggs was described by Ren [52] based on SEM observations. Okuda and Uchida [68] reported a nymph on mammal faeces with a forwardly pointed rostrum, but whether it was feeding on the juice of the faeces or preying on maggots, could not be confirmed.

**Remarks.***Bardesanes signatus* was described based on an unspecified number and sex of specimens (syntypic) from “Burma; Karennee” (=Kayah, Myanmar) [36]. A dorsal habitus and a lateral view of the head and prothorax were illustrated by Distant [37], along with a redescription of the species. One male syntype (Figure 3A–C) deposited in the BMNH and presumed to be the specimen illustrated by Distant [37] is here designated as the lectotype of this species. As *Bardesanes* is hereby considered a junior subjective synonym of *Argolis* (see discussion below), this species is transferred to the latter genus as *A. signata* (Distant, 1909), **comb. nov.**

The type material of *Caunus noctulus* consists of one male holotype and six paratypes (three males and three females, including one treated as the “allotype”) [27]. The holotype (Figure 3D–F) and three paratypes (one male and two females, including the “allotype”) deposited in IZAS were examined during this study. Hsiao [27] distinguished his new species from *B. signatus* by the following morphological characters (in Chinese): anterolateral angles of pronotum prominent; mid femur longer than fore femur, both not curved; antennal scape relatively short, distinctly shorter than pedicel. A reexamination of the type materials of both species concluded that they were conspecific. The antennal scape of the lectotype of *B. signatus* is clearly shorter than the pedicel but ambiguously described as “subequal in length to the second joint [= pedicel]” by Distant [36]; the minor differences in the shape of the anterolateral angles of the pronotum are considered as minor individual variations; the seemingly curved fore and mid femora of the lectotype of *B. signatus* are perhaps due to a deformity during the individual development. Therefore, we propose the following new subjective synonymy: *Argolis signata* (Distant, 1909), **comb. nov.** = *Caunus noctulus* Hsiao, 1977, **syn. nov.**

## 4. Discussion

### 4.1. Sexual Dimorphism in Argolis

Many subfamilies of Reduviidae are known to exhibit sexual dimorphism, and the morphological divergences between male and female of a certain species are sometimes extraordinary [74,75,76,77]. Limited and extreme sexual dimorphism have been documented within Stenopodainae as well [7,78,79] and sexual dimorphism of *Argolis* was described in Villiers [7,8], Hsiao [27] and Cao et al. [6]. The male and female of *Argolis* show differences in general habitus and several morphological character states summarized in Table 1.

Some of the above characters were previously utilized to separate *Argolis* and *Caunus* [10,18,20,22], but Schouteden [11] first recognized that such differences were attributed to sexual dimorphism, and he therefore synonymized the two genera. Likewise, characters related to sexual dimorphism affected species-level classification within *Argolis*. For instance, *A. farinator* was originally described from the female, while the male was subsequently described as *N. typica* (see above under *A. farinator*). In order to avoid misidentification caused by sexual dimorphic characters, both sexes of the two Asian species of *Argolis* are redescribed in this study. However, some of the 14 described species from the Afrotropical Realm are still known from a single sex, and their identities need to be verified in future studies.

### 4.2. Evaluation of Bardesanes and Neoklugia

Distant [36] treated his genus *Bardesanes* as “a genus to be placed after *Caunus* in the British Indian enumeration”, but he did not conduct a morphological comparison of these two genera. Livingstone and Ravichandran [29] included these two genera in their key to the South Indian genera of Stenopodainae. They used the morphology of the antennal scape to separate *Caunus* (now *Argolis*) and *Bardesanes*, but this character is sexually dimorphic as discussed above and therefore not appropriate for genus-level classification. Based on a redescription of the male of *A. farinator* (misidentified as *B. signatus*), Chandra et al. [47] listed four characters to differentiate *Bardesanes* and *Caunus*. We examine the lectotype (here designated) of *B. signatus* (the type species of *Bardesanes*) and make the following comments: (1) Chandra et al. [47] mentioned that the fore femur is spined in *Bardesanes* but unarmed in *Caunus*; this is not true because the fore femur of *B. signatus* only has some small setigerous tubercles on its ventral surface but no spines or denticles. (2) The visible labial segment I is “little longer than” segment II in *Bardesanes*, while in *Caunus* they are subequal in length [47]; based on the examination of a series of specimens of the two Asian species, we do not find clear differences in the length of the two basal labial segments. (3) The antennal scape is nearly as long as the pedicel in *Bardesanes* but shorter than the pedicel in *Caunus* [47]; this character was inaccurately described by Distant [36,37], because the pedicel of the lectotype of *B. signatus* is clearly longer than the scape; the pedicel is also more or less clearly longer than the scape in other conspecific specimens we have examined in this study; in the measurements provided by Chandra et al. [47], the pedicel of their specimen is significantly shorter than the scape, but this is the opposite of the state shown in their photo (Chandra et al., 2013: figure B [47]). (4) The apical process of the scutellum is described as “moderately curved upwards” in *Bardesanes* and “obliquely erect” in *Caunus* by Chandra et al. [47]; such descriptions are ambiguous and could not effectively distinguish the two genera; the apical process of the scutellum is more or less raised in our examined specimens, although it is weaker in females than in males; this is a minor individual variation and cannot be used to separate different genera. Consequently, *Bardesanes* could not be clearly distinguished from *Argolis* by morphological characters.

When describing *Neoklugia*, Distant [38] compared it with *Oncocephalus*. Most of the morphological characters listed by him match those of *Argolis*, except that fore femur is “only slightly or moderately incrassated, with a single series of slender short spinules beneath”, and hind leg “with the femora and tibiae about or almost of equal length” [38]. The examination of the type material of *N. typica* (the type species of *Neoklugia*) reveals that these two characters were inaccurately described by Distant [38]: the fore femur is not thickened and only has some small setigerous tubercles on its ventral surface; the hind tibia is significantly longer than the hind femur. Since *N. typica* is also confirmed as the male of *A. farinator* (see above under *A. farinator*), there seems no reason for maintaining *Neoklugia* as a distinct genus based on morphological characters.

In conclusion, we propose the following new subjective synonymies: *Argolis* Stål, 1861 = *Bardesanes* Distant, 1909, **syn. nov.** = *Neoklugia* Distant, 1919, **syn. nov.** *Argolis* (as *Caunus*) was usually confused with *Bardesanes* in some previous studies [46,47,73], and *Neoklugia* was almost never cited since its original description. Such confusion and neglect largely stem from the unclear identities of the two Asian species of *Argolis* and the resulting misidentifications. In the present study, the two Asian species of *Argolis* are redescribed and illustrated. In order to stabilize the nomenclature regarding *C. farinator*, *B. signatus* and *N. typica*, lectotypes of these species are designated. The differences between the two Asian *Argolis* species are summarized in Table 2.

### 4.3. Systematic Relationships of Argolis

*Argolis* has long been considered to be closely related to *Oncocephalus* [12,19,26,27,33]. These two genera share a similar general habitus and the following morphological characters: head subcylindrical; eye reniform in lateral view, nearly touching (♂) or far removed (♀) in ventral view; ocellus elevated; scutellum with apical process, horizontal or slightly curved upwards; tarsal formula 3-3-3; fore and mid tibiae lacking fossula spongiosa; hemelytron with hexagonal cubital cell. These morphological similarities likely indicate a close phylogenetic relationship between *Argolis* and *Oncocephalus*, and it is also possible that *Argolis* is a specialized lineage within a more extensive *Oncocephalus*. A cladistic analysis with samples of both genera is needed to evaluate these possibilities.

*Argolis* can be distinguished from *Oncocephalus* in its current sense by the nearly straight labium (vs. distinctly curved in *Oncocephalus*) and the slender and ventrally unarmed fore femur (vs. strongly thickened with one or two rows of spiniferous tubercles in *Oncocephalus*).

### 4.4. Distribution of Argolis

*Argolis* has a disjunct distribution in the Old World, with 14 species occurring in sub-Saharan Africa, including Madagascar, and the other two distributed in Asia (Table 3). The two Asian species exhibit geographic isolation.

*Argolis farinator* seems to be endemic to the Indian subcontinent, with most examined specimens from southern India (Figure 10). Our study extends the distribution of the species westward to Malir (southern Pakistan) and northward to Almora (northern India) and adds a new record for the species on the southern slopes of the Himalayas.

*Argolis signata***comb. nov.** is distributed in Southeast and East Asia, from the Indochinese Peninsula through southern and eastern China to the Japanese Archipelago, but two examined specimens were collected from Pedong (northern India) on the southern slopes of the Himalayas (Figure 10). Few specimens were collected on the Indochinese Peninsula, but further collecting could increase its distribution range in this area. The distribution of this species in China is mainly south of the Yangtze River, but it is also found more northerly in Shandong. In Japan, this species has been recorded in Honshu, Kyushu, Shikoku, and Tsushima Islands, but most collection records are concentrated in the Kanto Region [68,69]. This species was recorded in Henan, China [6] and the Korean Peninsula [35], but no voucher specimen from these localities could be examined during this study.

The biogeographic background of the distribution of *Argolis* is unclear. Previous phylogeographical studies have suggested that the Afrotropical, Malagasy, and Oriental reduviid faunas are closely related, and transoceanic dispersal has happened among these regions [84,85]. Whether this hypothesis is also appropriate for *Argolis* requires testing in future studies. In the case of the Asian fauna, the Indo-Myanmar Mountain ranges may have been a geographical barrier between *A. farinator* and *A. signata*, while the Japanese populations of *A. signata* are probably more closely related to those in southeastern China, but the demographic history of the genus in East Asia needs to be further analyzed.

## Figures and Tables

**Table 1:** Sexual dimorphic characters of *Argolis*.

	Male	Female
General habitus	Elongate oval	Subfusiform
Postocular region of head	Gradually converging posteriorly	Nearly parallel-sided or only weakly converging posteriorly
Eye	Large, nearly touching each other in ventral view	Small, far removed from each other in ventral view
Ocellus	Distinctly elevated	Slightly elevated
Antennal scape	Longer than head, straight, strongly hairy	Shorter than head, curve, finely hairy
Antennal pedicel	Strongly hairy	Finely hairy
Pronotum	Wider than long, anterior lobe distinctly shorter than posterior lobe	Longer than wide, anterior lobe slightly shorter than posterior lobe
Humeral angles of pronotum	Acute, slightly to distinctly protruding laterally	Blunt, angulated or acute, weakly protruding laterally
Hind femur	Reaching apex of abdomen	Not reaching apex of abdomen
Hemelytron	Reaching or slightly surpassing apex of abdomen	Far removed from apex of abdomen
Abdomen	Elongate oval	Subfusiform
Intersegment suture between abdominal sternites VI and VII	Widely curved anteriorly	Sharply incised anteriorly at midpoint

**Table 2:** Diagnostic characters of *Argolis farinator* and *Argolis signata* comb. nov.

	*Argolis farinator*	*Argolis signata*, comb. nov.
General body color	Yellowish brown	Dark brown
Setigerous tubercles on head and prothorax	Prominent	Minute
Ratio of anteocular and postocular regions of head	More than 2.7 times	Less than 2.2 times
Tubercles on posterior head margin	Prominent	Blunt
Granulations on disc of pronotum	Two distinct pairs	Two indistinct pairs
Granulations on lateral margin of pronotum	Present	Absent
Coloration of femur	Fore and mid femora yellowish brown, hind femur dark brown	Brown to dark brown
Coloration of tibia	Uniformly yellowish brown	Yellowish brown with basal and subbasal annuli
Coloration of hemelytron	Coriaceous portion yellowish brown, membrane pale greyish brown	Coriaceous portion brown, membrane dark greyish brown
Cubital cell of hemelytron	Shorter than half of length of apical external cell	Longer than half of length of apical external cell
Pygophore	Finely expanded in dorsal view, ventral surface not emarginate subapically in lateral view	Distinctly expanded in dorsal view, ventral surface strongly emarginate subapically in lateral view
Paramere	Relatively slender, with a short subapical keel	Relatively stout, with a wide subapical keel enclosing an arc with apex of paramere
Phallus	Basal plate arms gradually divergent apically, struts simply curved at bases	Basal plate arms widely separated at base and slightly convergent apically, struts bisinuate at bases
Abdominal tergite IX in female	Apical half relatively broad	Apical half relatively narrow
Valvula II	Apically acute	Apically blunt

**Table 3:** Described species of *Argolis* and their known distribution (AF: Afrotropical; OR: Oriental; PA: Palaearctic).

Species	Distribution	References
*Argolis acuta* Schouteden, 1951	AF: DR Congo	[80]
*Argolis bergrothi* Schouteden, 1902	AF: DR Congo	[11]
*Argolis calabarensis* (Stål, 1858)	AF: Benin, DR Congo, Guinea, Nigeria, Togo	[7]
*Argolis capensis* (Stål, 1855)	AF: South Africa	[81]
*Argolis dolichomera* (Reuter, 1882)	AF: South Africa	[12]
*Argolis farinator* (Reuter, 1882)	OR: India, Pakistan, Sri Lanka	Present study
*Argolis lamtoensis* Villiers, 1965	AF: Côte d’Ivoire, Guinea	[82]
*Argolis meloui* Villiers, 1948	AF: DR Congo, Niger, Senegal, Tchad	[7]
*Argolis moniliata* Miller, 1950	AF: Uganda	[13]
*Argolis nigrofasciata* Villiers, 1963	AF: Guinea	[83]
*Argolis pedestris* Miller, 1952	AF: South Africa	[14]
*Argolis proxima* Schouteden, 1902	AF: Congo, DR Congo	[7,11]
*Argolis seyrigi* (Villiers, 1951)	AF: Madagascar	[8]
*Argolis signata* (Distant, 1909), comb. nov.	OR: China, India, Laos, Myanmar, Vietnam; PA: Japan	Present study
*Argolis ugandensis* Villiers, 1962	AF: Uganda	[15]
*Argolis villiersi* Schouteden, 1951	AF: Angola, DR Congo	[1,80]

## Data Availability

Not applicable.
